# The Effectiveness of Workplace Musculoskeletal Injury Risk Factor Screening Tools for Reducing Injury: A Systematic Review

**DOI:** 10.3390/ijerph20032762

**Published:** 2023-02-03

**Authors:** Richard Roberts, Teri Slade, Don Voaklander, Sebastian Straube, Liz Dennett, Carol Cancelliere, Christine Guptill, Linda Miller, Danielle Lemay, Melnard De Leon, Douglas P. Gross

**Affiliations:** 1 School of Public Health, University of Alberta, Edmonton, AB T6G 1C9, Canada; 2 Faculty of Rehabilitation Medicine, University of Alberta, Edmonton, AB T6G 2G4, Canada; 3 Division of Preventive Medicine, Department of Medicine, University of Alberta, Edmonton, AB T6G 2R3, Canada; 4 John W. Scott Health Sciences Library, University of Alberta, Edmonton, AB T6G 2R7, Canada; 5 Faculty of Health Sciences, Ontario Tech University, Oshawa, ON L1H 7K4, Canada; 6 School of Rehabilitation Sciences, University of Ottawa, Ottawa, ON K1H 8M5, Canada; 7 EWI Works, Edmonton, AB T6E 3N8, Canada; 8 Occupational Hygiene & Product Safety, Suncor Energy, Calgary, AB T2P 3E3, Canada; 9 Department of Physical Therapy, University of Alberta, Edmonton, AB T6G 2G4, Canada

**Keywords:** occupational health, musculoskeletal pain, cumulative trauma disorders, insurance, disability, compensation and redress, workers’ compensation, sick leave, ergonomics, employment

## Abstract

Introduction: Musculoskeletal injury (MSI) contributes to global health burdens. Effective MSI prevention is necessary. MSI risk factor screening tools can be used by employers to identify and mitigate occupational hazards. Rigorous synthesis of the effectiveness of these tools has not taken place. We synthesized literature on effectiveness of MSI risk factor screening tools for reducing injury through informing prevention interventions. Materials and Methods: A literature search of Medline, Embase, Cochrane Library (Trials), CINAHL, Scopus and PsycINFO databases was performed. Included studies required an analytic design, used an MSI risk factor screening tool to inform an intervention in a working-age population and reported an outcome of MSI development, injury or compensation/work absence. Data extraction and study quality rating (Downs and Black criteria) were completed. Studies were sub-categorized as having used a single MSI screening tool (single-tool) to inform an injury prevention intervention or involving multiple simultaneous screening tools (multiple-tool). Study outcomes were synthesized when possible. Results: Eighteen articles representing fourteen studies met our inclusion criteria. No high-quality studies were identified (maximum Downs and Black score of 19). Studies did not employ previously validated tools but instead, typically, those purpose-built for a single use. The results were inconsistent both when using tools alone and in combination with other tools. Outcome measure heterogeneity precluded meaningful meta-analysis. Conclusions: There is limited evidence regarding use of MSI risk factor screening tools for preventing injury. Rigorous studies that utilize previously validated tools are needed.

## 1. Introduction

Musculoskeletal injuries (MSI) are among the largest contributors to the global burden of pain, disability and work loss [1]. The prevalence of MSI is increasing worldwide, most notably among low- and middle-income countries [2]. We lack a unified international-level strategy to prioritize their treatment, as exists more generally for communicable diseases [2]. Given these substantial burdens and alongside current treatment barriers, there is a definitive need for strategies that mitigate MSI symptoms or prevent incident MSI (primary prevention) [3]. The latter strategy is especially important and can be enacted through targeted and effective interventions in populations that are most at risk of MSI. Workers exposed to physical loading in the workplace are a key population for these targeted approaches. Since 2000, occupational exposure causing neck and back pain has alone contributed nearly 14% of all occupational disability-adjusted life years globally [4]. Occupational health and safety regulations often have employers identify, assess and control or reduce occupational risk factors associated with MSI. Various MSI risk factor screening tools exist and are aimed at risk identification. These tools include, but are not limited to, questionnaires as well as observational criteria to identify types of workload risk—including intensity, frequency or duration of tasks [5]. A recent scoping review identified 19 different risk assessment tools, concluding that this was a “large number of observational assessment tools” [5]. In a North American context, MSI screening tools most commonly used by occupational health professionals across job sectors include the NIOSH Lifting Equation, Rapid Upper Limb Assessment (RULA) and Rapid Entire Body Assessment (REBA) [6].

Throughout this review, MSI risk factor screening tools are viewed in the context of informing interventions to prevent MSI and its effects. Previous research has reviewed the measurement properties of MSI risk factor screening tools, with varying reliability and validity reported [7]. Reliability appears to vary across items within individual tools and depends on rater experience [8]. However, the primary goal of using these tools is to reduce the risk and consequences of MSI in the workplace. To reduce risk of reported MSI, these tools typically inform use of specific interventions that directly address and mitigate the risks identified by the tool. In fact, several tools recommend application of specific interventions that are contingent on risk levels or scores identified by the tool. For example, the RSI QuickScan questionnaire is an MSI risk factor screening tool that establishes a risk profile for arm, shoulder and neck symptoms [9]. This information then informs tailored interventions based on the individual risk profile, with 16 possible interventions implemented based on a decision tree algorithm. The recommendations are thus an integral part of the tools’ scoring. Therefore, in the context of informing interventions, the effectiveness of an MSI screening tool depends on how accurately the tool identifies risk factors as well as how effectively it informs the implementation of (a) targeted prevention intervention(s). Despite the apparent breadth of MSI risk factor screening tools, some researchers have raised concerns about their utility and effectiveness [10,11]. MSI risk factor screening tools are typically developed using biomechanical, laboratory or consensus studies rather than through methodologically rigorous trials in actual work environments. Furthermore, rationales for adoption of industrial standards and threshold limits for workload exposures have been criticized as lacking rigor or transparency. Armstrong et al. recommend a solution: formal evaluation of these risk assessment procedures using the same techniques required for medical or public health standards [11].

Research is needed to assess the current scientific literature involving evaluation of the effectiveness of MSI risk factor screening tools for informing injury prevention interventions through a rigorous epidemiological lens. This will provide critically important information regarding whether these tools can successfully be used to prevent MSI and its consequences. Therefore, our research question was: “in working age adults, what is the effectiveness of MSI risk factor screening tools for preventing onset and consequences (i.e., pain, disability, quality of life, work loss and reduced productivity) of work-related MSI?”

## 2. Materials and Methods

This review followed Preferred Reporting Items for Systematic Reviews and Meta-Analysis guidelines [12]. The review protocol was registered with the International Prospective Register of Systematic Reviews (CRD42021232747).

A systematic literature search was carried out by a health sciences librarian (L.D.) in Medline via Ovid (1946–18 March 2021), Embase via Ovid (1974–18 March 2021), Scopus (searched 19 March 2021), CINAHL Plus with Full Text (via EBSCOhost) (1937–19 March 2021), Wiley Cochrane Central Register of Controlled Trials (CENTRAL) (searched 19 March 2021) and APA PsycINFO (1806–November Week 3, 2021) databases. Team members collaborated to develop a sensitive search strategy that utilized two approaches: (1) searching by the names of specific tools identified in a preliminary literature review or provided by stakeholders ([L.M., D.L.]), and (2) searching generically with combinations of subject headings and keywords pertaining to MSI, occupational settings and screening tools. The results of both approaches were limited to quantitative primary research studies only. The grey literature was not searched, which is a change from our protocol. After preliminary searching of the voluminous grey literature, it was determined that this searching would not result in rigorous evaluations, which was the focus of this study. Our definition of MSI was adapted from WorkSafeBC’s definition that encompasses injuries and disorders of muscles, tendons, ligaments, joints and soft tissues (nerve and vascular injury) [13]. For this study, we did not include generic search terms for vascular, nerve or vibration-induced injuries but did include specific search terms for carpal tunnel syndrome. The full search strategy is available (see Supplementary Material S1).

A PICOS (Population, Intervention, Comparison, Outcome, Study Design) framework was used for development of article inclusion criteria. Articles were required to have an English-language title and abstract and were eligible if they assessed:

P—a working-age population in a working environment (exclusion of pre-employment screening and studies in military populations);

I—applied an MSI risk factor screening tool using individual or workplace-related risk factors to prevent MSI injury and its related consequences (exclusion of studies reporting only measurement properties, such as predictive validity);

C—compared to other MSI prevention strategies that were not informed by an MSI risk factor screening tool;

O—reported on at least one primary outcome related to MSI development, injury or compensation/insurance claims (a variety of secondary outcomes were also examined, including workplace MSI risks and behaviors, as well as any self-reported MSI outcomes, such as pain, disability, discomfort, etc.);

S—utilized an analytic study design (i.e., randomized clinical trial; cohort, case-control study, quasi-experimental studies).

**Figure 1:**
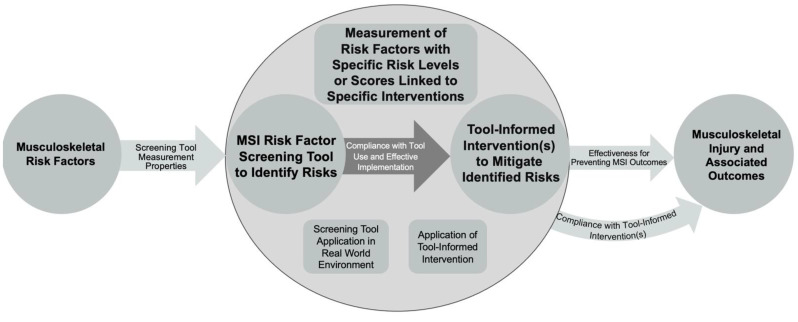
Logic map showing how musculoskeletal injury (MSI) risk factor screening tools inform interventions to affect MSI-associated outcomes.

We expected that MSI risk factor screening tools would affect injury outcomes by conditionally applying a specific prevention intervention based on risk levels or scores identified by the screening tool. Tool results inform implementation of a tailored intervention aimed at mitigating any identified risk factors. For example, if the tool identifies excessive physical loading as a risk, the recommendation may be to modify work tasks or activities to reduce risk or have workers undergo a fitness or strengthening program to increase their manual handling abilities. A variety of risk factor/intervention combinations are possible for both physical and mental risks at the workplace. This process is conceptualized in a logic map in Figure 1.

We made modifications to our review protocol prior to our analysis. Specifically, to capture all potentially relevant articles, studies did not require a minimum sample size to be included. Additionally, we clarified that eligible study populations must not have been identified as injured prior to study enrolment; thus, eligible outcomes became incident MSI, compensation claims or insurance claims. 

Following completion of the database search, article titles and abstracts were added to online review manager Covidence [14] and de-duplicated. Titles and abstracts were then independently screened for initial inclusion by research team members. If two research team members concluded that an article potentially met inclusion criteria, or that eligibility could not be ascertained from title and abstract alone, the full-text article was obtained. Disagreements at abstract stage were resolved first by consensus and then by a senior research team member if any remained unresolved. 

Full-text articles were independently assessed for eligibility by a smaller subset of the research team. Articles had to be deemed eligible by two team members, and disagreements at full-text stage were resolved by consensus prior to or after consulting the third team member. Articles for which consensus was not reached at the full-text stage were provided to the entire research team for discussion. Additional articles were identified directly for full-text eligibility screen through citation searching of included articles and systematic reviews identified during screening. One article was identified as a subsequent analysis of a study population from an article included at full-text stage and was retrieved for full-text eligibility screening. 

A standardized spreadsheet was used for data extraction of included articles. One reviewer performed the initial data extraction, with verification by a second reviewer. Extracted article data included study design, study setting and context, participant characteristics, MSI screening tool descriptions and alternative treatments of study arms, outcome measure descriptions and reported outcome results. Effect estimates were presented where possible.

Included articles were synthesized depending on their method of MSI risk factor screening tool application. The first category of “single-tool” articles contains studies that, in at least one study arm, applied a single MSI screening tool to inform an intervention in isolation from any other additional screening tools, assessments or interventions. These study designs provide the most direct assessments of MSI screening tool effect. The second category of “multiple-tool” articles applied one or more MSI risk factor screening tools in combination with other assessments and interventions (that may or may not have been informed by the screening tool of interest). For this latter group of articles, it was deemed that the causal effect of any single MSI screening tool use could not be meaningfully isolated from the causal effect of distinctly separate but concurrently applied assessments and associated interventions. Consultation with community partners indicated that prevention interventions within industry contexts are most often pragmatically applied in “multiple-tool” situations. 

The Downs and Black (D&B) quality assessment checklist was used to assess included article quality [15]. The quality assessment checklist contains 27 questions assessing quality of reported material, internal validity stemming from selection bias, information bias and confounding as well as external validity and study power [15]. The checklist is appropriate for quasi-experimental, cohort and randomized control trial (RCT) study designs, allowing simple comparison between a plurality of study methodologies. The Downs and Black score was assigned out of a total possible 28 points for each article. Score interpretation has previously used quality bands of excellent (>25), good (20–25), fair (15–19) and poor (≤14) article quality [16].

This review follows principles of best evidence synthesis and incorporates components of Synthesis Without Meta-analysis reporting guidelines, the latter of which is intended to complement PRISMA reporting guidelines [17,18]. All included articles of medium quality or higher (D&B of fair or better) were retained for narrative synthesis. Study outcome categorizations were adapted from the original protocol and included musculoskeletal discomfort, work absence, health resource utilization, changes to workplace behaviour, self-assessed health status, workplace-related MSI and claims cost. Outcome metrics were standardized using effect direction, as recommended by Boon and Thomson (direction reported if >70% of categorized study outcomes had similar direction of effect), with consistency of evidence for these outcomes assessed using an effect direction plot adapted from the same authors [19]. A sign test was not performable for assessment of outcome heterogeneity due to too few articles. An algorithm for evidence level (strong to insufficient) was adapted from the Institute for Work and Health [20] (Table 1). Any materials used in the review are available from the authors.

## 3. Results

**Figure 2:**
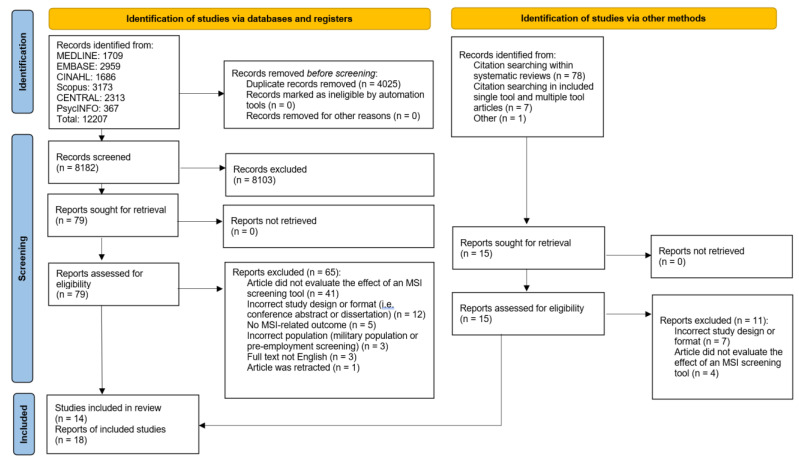
PRISMA 2020 flow diagram for new systematic reviews which included searches of databases, registers and other sources [12].

The initial database search yielded 12,207 results, and 4025 duplicates were removed; 8182 articles were screened for potential eligibility, of which 79 full-text articles were reviewed for inclusion. Percent agreement during abstract screening ranged from 88% to 100%, and all discrepancies were resolved through consensus. Fourteen articles met the inclusion criteria following full-text review and were included for analysis. Citation searching from the included articles, key systematic reviews and incidental related articles yielded 15 articles that were retrieved for full-text analysis. Four articles were retained from this second identification group. In total, 18 articles were included for quality assessment and data extraction. Most articles excluded at the full-text stage did not evaluate the effect of an MSI risk factor screening tool (see Figure 2).

Article quality appraisal was applied using the Downs and Black criteria (Supplementary Table S2). Articles of at least medium quality (Downs and Black score band of fair; see Table 1) were retained for narrative synthesis. No high-quality articles were identified. 

### 3.1. Characteristics of Single-Tool Articles

Five single-tool articles representing four studies were identified and retained following quality assessment. All five articles were scored as medium-quality, meeting at least half of the methodological criteria [9,21,22,23,24]. Positives included reporting of most necessary information, real-world study environments, reasonable intervention compliance, low likelihood of influence from participants lost to follow-up and typically adequate power. Negatives included poor reporting of potential adverse events or characteristics of participants lost to follow-up, poor generalizability from participant selection and sampling methodology, mixed accuracy of outcome measures and some incomplete adjustment for potential confounders.

Supplementary Table S3 (upper half) summarizes the characteristics of the retained single-tool studies, all of which are RCTs. One study, reported in two articles, assessed an MSI screening tool and tool-guided interventions based on occupational health [9] and economic [24] outcomes. The study participants were either computer users [22,23] or part of a general working population [9,21,24]. Participants were followed anywhere from 2 weeks to 2 years [21,22] following tool use, and screening tool arm sample sizes ranged from 35 to 1374 participants [21,23]. MSI risk factor screening tools were used in these studies to inform a variety of work modifications, including administrative controls and physical hazard elimination [21], ergonomic workplace adjustment [22,23] and a multicomponent intervention program [9,24]. Data sources for outcome measures included self-report questionnaires [9,22,23,24], daily symptom diaries [23] and company-provided occupational data [9,21,24]. Other comparator arms included tool-assisted risk assessment but withholding tool recommendations until completion of follow-up [9,22,24] and providing a variety of general [23] or specific [21] occupational health information to participants. The computer-user studies focused heavily on measurements of musculoskeletal discomfort [22,23] while also including some behavioural change measures. The general working population studies more frequently reported measures of work absence [9,21,24], and one included resource utilization measures [24].

### 3.2. Characteristics of Multiple-Tool Articles

Thirteen multiple-tool articles were identified [25,26,27,28,29,30,31,32,33,34,35,36,37], and seven, representing five studies, were retained following quality assessment [26,27,28,30,35,36,37]. All seven retained articles were scored as medium-quality. Compared to the single-tool articles, the multiple-tool articles described confounding variables and patients lost to follow-up less frequently and did not provide a priori indicators of follow-up articles for related same-study articles. The multiple-tool study populations did, however, have higher representativeness of their source populations.

Supplementary Table S3 (lower half) summarizes the characteristics of the retained multiple-tool studies. One study encompassed three follow-up articles [26,27,28] published from 2002 to 2005, with an original 2001 article not retained due to poor article quality [25]. Study design variety was larger in these studies, with three quasi-experimental study designs [26,27,28,30,36] and two RCTs [35,37]. Participants in the studies included health workers from Canada [30] and Australia [26,27,28], construction workers from the Netherlands [35], foundry workers from Italy [36] and farmers from the United States [37]. Follow-up was typically longer than included study counterparts—12 months at minimum. The range of sample sizes was comparable with the included studies. Data sources for the retained multiple-tool studies included workplace-associated records [26,27,28,35,36], insurance compensation documents [26,27,28], regional occupational health records [30] as well as self-report forms [35,37] and standardized phone calls [37]. Five studies reported count or rate outcomes of workplace-associated MSI [26,27,28,30,36,37], all but one reported a measure of work absence [26,27,28,35,36,37], one reported a measure of musculoskeletal discomfort [35] and three reported a measure of claims cost [26,27,28,37]. One study reported on measures of other healthcare utilization [37] and another reported on self-assessed health status [35].

Six articles were scored as poor quality and are not characterized in this paper beyond their quality appraisals [25,29,31,32,33,34]. Compared to the retained articles, these poor-quality articles less frequently reported on study characteristics, were significantly less representative of their source populations, did not necessarily recruit comparable groups for screening tool use and control groups, did not adequately adjust for differing participant follow-up time or confounding by other means and used less valid outcome measurement instruments.

### 3.3. Synthesis of Included Study Results

Table 2 presents the effect direction plot showing consistency of outcomes for the included studies. In total, seven outcome categories were provided from the included studies—musculoskeletal discomfort, work absence, health resource utilization, work behavior modification, workplace-associated MSI, claims cost and self-rated health status. The results according to these outcomes are shown below.

No high-quality studies are present in the analysis, and each study utilizes a different MSI risk factor screening tool. Therefore, there is insufficient evidence to determine the effect of any specific MSI risk factor screening tool on any of the previously identified outcome categories.

### 3.4. Effects on Musculoskeletal Discomfort

Three medium-quality single-tool studies show either conflicting evidence [22,23] or no change [9,24] in musculoskeletal discomfort measures following their respective MSI-risk-factor-screening-tool-guided interventions. One medium-quality multiple-tool study shows no change [35] in musculoskeletal discomfort measures following use of an MSI risk factor screening tool as an intervention component. Therefore, there is limited evidence that MSI risk factor screening tools either do not affect or inconsistently affect musculoskeletal discomfort when used by themselves and insufficient evidence of their effect on musculoskeletal discomfort when used in combination with other interventions.

### 3.5. Effects on Work Absence

Two medium-quality single-tool studies show no change [9,21,24] in work absence measures following their respective MSI-risk-factor-screening-tool-guided interventions. Three medium-quality multiple-tool studies show no change in work absence measures [35,36,37] and one medium-quality study shows a decrease in work absence measures [26,27,28] following use of an MSI risk factor screening tool as an intervention component. Therefore, there is limited evidence that MSI risk factor screening tools either do not affect or inconsistently affect work absence, both when used by themselves or in combination with other interventions.

### 3.6. Effects on Health Resource Utilization

One medium-quality study shows no change [9,24] in measures of health resource utilization following an MSI-risk-factor-screening-tool-guided intervention. No included multiple-tool studies assessed health resource utilization outcomes following use of an MSI risk factor screening tool as an intervention component. Therefore, there is insufficient evidence regarding the effect of MSI risk factor screening tools on health resource utilization, both when used by themselves or in combination with other interventions.

### 3.7. Effects on Workplace Behaviour

One medium-quality study shows conflicting evidence [22] in measures of workplace behavior modification following an MSI-risk-factor-screening-tool-guided intervention. No included multiple-tool studies assessed workplace behavior modification outcomes following use of an MSI risk factor screening tool as an intervention component. There is insufficient evidence regarding the effect of MSI risk factor screening tools on work behavior modification, both when used by themselves or in combination with other interventions.

### 3.8. Effects on Workplace-Associated MSI

No included single-tool studies assessed workplace-associated MSI outcomes following an MSI-risk-factor-screening-tool-guided intervention. Two medium-quality multiple-tool studies show decreases in workplace-associated MSI [26,27,28,36], another shows an increase in workplace-associated MSI [30] and another shows no change [37] following use of an MSI risk factor screening tool as an intervention component. Therefore, there is insufficient evidence regarding the effect of MSI risk factor screening tools on workplace-associated MSI when used by themselves and mixed evidence when used in combination with other interventions.

### 3.9. Effects on Claims Costs

No included single-tool studies assessed measures of claims cost following an MSI-risk-factor-screening-tool-guided intervention. One medium-quality multiple-tool study shows decreases in claims cost [26,27,28] and another medium-quality multiple-tool study shows no change in claims cost [37] following use of an MSI risk factor screening tool as an intervention component. Therefore, there is insufficient evidence regarding the effect of MSI risk factor screening tools on claims costs when used by themselves and mixed evidence when used in combination with other interventions.

### 3.10. Effects on Self-Rated Health Status

No included single-tool studies assessed measures of self-rated health status following an MSI-risk-factor-screening-tool-guided intervention. One medium-quality multiple-tool study shows no change [35] in measures of self-rated health status following use of an MSI risk factor screening tool as an intervention component. Therefore, there is insufficient evidence for use of MSI risk factor screening tools on self-rated health status both when used by themselves or in combination with other interventions.

## 4. Discussion

The current evidence is insufficient to characterize the effect of MSI risk factor screening tool use on relevant MSI outcomes when used by themselves. The available evidence demonstrates an inconsistent effect of screening tool use on musculoskeletal discomfort and work absence. When used in combination with other tools and interventions in the context of a broader injury prevention program, there is mixed evidence for the effect of MSI risk factor screening tools on workplace-associated MSI and claims costs. For more certain conclusions on the utility and real-world effectiveness of MSI risk factor screening tools, high-quality randomized controlled trials should be conducted examining the impact of the currently available tools on MSI injury and related outcomes. If used in workplace settings, MSI risk factor screening tools should be one component of a broader MSI risk mitigation strategy.

This study utilized rigorous epidemiological data synthesis methods to assess the current state of the scientific literature regarding the effect of using MSI risk factor screening tools to inform injury prevention interventions on important outcomes, such as MSI and related claims and resource utilization. In total, 18 articles representing 14 studies met the article inclusion criteria dictated in the final protocol. Of these 18 articles, only 12 met the minimum quality criteria for retention in the literature synthesis. Of these twelve articles, only five—representing four studies and containing no overlap in screening tools used—used an MSI risk factor screening tool to guide an intervention in a manner that enabled meaningful isolation of the effect of the tool as compared to the effect of other distinctly separate but concurrent tools and interventions. Despite plausible isolation of the effects of these remaining screening tools, outcome measures were too heterogeneous to allow effect size data pooling; rather, the highest level of evidence that could be gleaned from the current literature is, overall, whether screening tools were or were not associated with a positive health effect for specified outcome measure categories.

There are numerous supplementary findings from this systematic review. First, none of the named tools from the preliminary database search that were identified as commonly used (e.g., NIOSH lifting equation, RULA, REBA) were found to have been evaluated rigorously beyond their own validation studies. This literature shows that, instead, MSI risk factor screening tools are, in practice, typically purpose-built or adopted from local occupational health centres. Occupational health and safety professionals designing these novel tools would see minimal examples supporting use of specific screening tools in the literature and instead may base their designs on international standards for biomechanical risk factors, which themselves are not definitively robust [11]. Any documentation of a high-quality, targeted and real-world application using a previously validated tool would significantly strengthen the state of the current MSI risk factor screening tool literature, especially if such studies also employ clearly defined, replicable outcome measures. In time, tool use resulting in more consistent positive health effects could be identified, adopted and refined.

Second, there is a distinct difference between the characteristics of single-tool and multiple-tool studies, the former group requiring that the effect of a single tool be identifiable. Notably, the selected study sample in multiple-tool studies was more consistently representative of its source population. These studies used a more pragmatic approach to screening and intervention and may better reflect actual practice, where, often, numerous assessment tools and potential interventions are simultaneously introduced in an attempt to improve some aspect of MSI. One conclusion from this finding is that MSI risk factor screening tool use is commonly only one component of a broader MSI risk mitigation strategy. It remains unclear how the effect of MSI screening tools changes with different types of concurrent interventions. This is an additional research avenue made clear from the results of the current systematic review.

This study provides, to the authors’ knowledge, the first systematic review specifically assessing the effects of MSI risk factor screening tools in actual work environments for informing MSI prevention programs. The strengths of the study included use of a robust database search strategy created through collaboration with an experienced health sciences librarian, use of up-to-date guidelines on systematic review structure and reporting and involvement of multiple stakeholder groups to provide guidance on practical needs of the occupational health and safety industry. Previous research synthesis has focused instead on the variety of available MSI risk factor screening tools [5], the effect of overall occupational health and safety interventions on preventing similar categories of MSI outcomes [20] and on use of clinical decision support tools to identify useful interventions for already injured patients with disabling musculoskeletal disorders [38]. However, the conclusions from this review show similarities to those from the occupational health and safety intervention review: both identify significant areas of evidence limited in certainty by a lack of high-quality literature, albeit the latter involving a substantially larger sample of 36 studies [20]. Considering the wide array of available MSI risk factor screening tools, this lack of data may point to the possibility of missed MSI screening tool use in the grey literature, which was not searched. This constitutes a limitation to our methods, yet we are confident that we located the highest-quality peer-reviewed articles in this research area. Another limitation of the current study was that we did not evaluate the large body of research examining the measurement properties (i.e., reliability and validity) of the MSI screening tools. However, this has been examined in previous reviews [7,8] and our focus on trials examining the real-world impact of MSI screening tools is novel.

## 5. Conclusions

Overall, there is a small quantity of insufficient research and limited evidence regarding use of MSI risk factor screening tools for informing injury prevention interventions. For more certain conclusions on the utility and effectiveness of MSI risk factor screening tools, high-quality research on the currently available tools is necessary.

## Figures and Tables

**Table 1:** Decision algorithm for level of evidence. Adapted from the Best Evidence Synthesis Guidelines used by Kennedy et. al [20].

Evidence Level	Minimum Study Quality according to Downs & Black (D&B) Rating	Minimum Study Quantity	Consistency
Strong	High (D&B score band of good or better)	3 or more studies	Agreement of effect direction in 3 high quality studies. For ≥3 studies, at least 75% of high- and medium-quality studies agree in effect direction
Moderate	Medium (D&B score band of fair)	2 high quality OR 2 medium quality and 1 high quality	Effect directions from 2 high quality studies agree OR effect directions from 2 medium studies and 1 high quality study agree. For ≥3 studies, effect direction agreement in more than 66% of studies
Limited	Medium (D&B score band of fair)	1 high quality OR 2 medium quality OR 1 medium and 1 high quality	Effect directions from 2 medium- or high-quality studies agree. If ≥2 studies, more than 50% of medium and high-quality studies agree
Mixed	Medium or high D&B score bands	2 studies	Effect directions from medium and high-quality studies are contradictory
Insufficient	No high quality, only 1 medium quality, any number of low (score band of poor) quality studies		

**Table 2:** Effect direction plot for retained included studies.

	Single-Tool Studies				Multiple-Tool Studies				
Study	Frost 2007 [21]	Ho 2014 [22]	Ketola 2002 [23]	Speklé 2010 [9,24] (RCT and economic analysis)	Carrivick 2002, 2005 [26,27,28]	Craib 2007 [30]	Porru 2017 [36]	Rautainen 2004 [37]	Oude Hengel 2013 [35]
MSI Risk Factor Screening Tool	Danish working environment regulations	DSE RAM System	Ergonomic checklist for VDU work	RSI QuickScan	Manual handling checklist (+ multiple interventions)	Custom-checklist-based screen (+ multiple interventions)	Ad hoc risk assessment checklist (+ multiple interventions)	Certified Safe Farm checklist (+ multiple interventions)	QuickScan questionnaire (+ multiple interventions)
Musculoskeletal discomfort		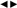	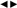	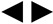					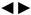
Work absence	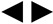			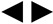	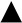		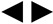	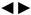	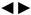
Health resource utilization				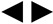				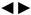	
Work behavior modification		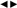							
Workplace-associated MSI					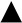	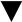	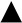	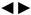	
Claims cost					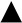			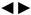	
Self-rated health status									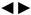

In this plot, upward arrow ▲ indicates positive health effect, downward arrow ▼ indicates negative health effect and sideways arrow ◄► indicates no change/mixed/conflicting findings. Final sample size in intervention group: large arrow▲ indicates >300, medium arrow ▲ indicates 50–300 and small arrow ▲ indicates <50. Study quality (Downs and Black) for all included studies was medium.

## Data Availability

Data available upon request from the authors.

## Supplementary Materials

The following supporting information can be downloaded at: https://www.mdpi.com/article/10.3390/ijerph20032762/s1, Supplementary Material S1: Search Strategy, Table S2: Downs and Black study quality assessment scoring for eligible articles, prior to removal due to poor quality, Table S3: Characteristics of included studies: Characteristics included design, setting, description of MSI screening tool, study arm description, outcome measurements, and results.
