# Supplementary material for: The Effectiveness of Workplace Musculoskeletal Injury Risk Factor Screening Tools for Reducing Injury: A Systematic Review
Source: Int J Environ Res Public Health. 2023 Feb 3;20(3):2762. doi: 10.3390/ijerph20032762 (PMC9916206; doi:10.3390/ijerph20032762)
Supplement: Supplementary file 1 [file ijerph-20-02762-s001.zip › ijerph-2130586-supplementary.pdf]

Supplementary Material S1: Search Strategy

Ovid MEDLINE(R) ALL 1946 to March 18, 2021

Date searched: March 19, 2021

Results: 1709

1. Accidents, Occupational/ or workers' compensation/ or Occupational Health/ or Occupational Injuries/ or Occupational Health Services/

2. employment/ or Work/ or work performance/ or Workplace/

3. exp *health personnel/

4. industry/ or beauty culture/ or forestry/ or health care sector/ or laundering/ or manufacturing industry/ or construction industry/ or chemical industry/ or exp "extraction and processing industry"/ or textile industry/ or power plants/

5. occupational groups/ or administrative personnel/ or farmers/ or government employees/ or laboratory personnel/ or metal workers/ or miners/

6. (white-collar or blue-collar or shift-worker* or attorney* or lawyer* or miners or mining-industry or office-worker* or desk-worker* or secretarial-worker* or administrative-assistant* or secretaries or manual-labourer* or manual-laborer* or physical-labourer* or physical-laborer* or farmworker* or ((warehous* or construction or farm or agricultur* or fast-food or food-service or grocery or store or retail or maintenance) adj3 (work* or employ* or job* or labor* or labour* or industry)) or steelworker* or iron worker* or ironworker* or mill-worker* or railway-worker* or vehicle-operator* or equipment-operator* or machinery-operator* or transit-operator* or bus-driver* or government-employees or hairdresser* or barber* or esthetician or child-care-providers or child-care-workers or housecleaners or janitor*).mp.

7. (((hospital adj (worker* or employee*)) or healthcare aide* or surgeon* or doctor* or physician* or general practitioner* or nurse* or dentist* or hygienist* or masseur* or masseuse or massage therapist*) and (pain or safety or safe or unsafe* or accident* or injur* or re-injur* or ergonomic or postur* or msk or strain or musculoskeletal)).ti.

8. (occupation* or (work* not social work) or employ*).jw.

9. ((work* not working-memory) or occupation* or employ* or job or jobs).ti,kf.

10. (work-related or job-related or employment-related or working-conditions or work environment* or workplace* or work place* or worksite* or work site* or jobsite or job site or "at work" or "on the job" or "while working" or Work-related or work* compensation).mp.

11. (Accidents/ or Accident Prevention/ or Safety/) and (employ* or work* or occupation* or job* or industr* or labourer* or laborer*).mp.

12. ((safety or safe or safely or unsafe* or accident* or hazard* or injur* or re-injur*) adj10 (employ* or (work* not working-memory) or occupation* or job* or industr* or labourer* or laborer*)).mp.

13. occupational disease*.mp.

14. (job-specific or work-specific or preemployment or pre-employment or work assessment or job assessment or jobfit or job matching).mp.

15. or/1-14

16. Musculoskeletal Diseases/ or joint instability/ or joint loose bodies/ or synovitis/ or ischemic contracture/ or contracture/ or dupuytren contracture/ or exp arm injuries/ or exp back injuries/ or contusions/ or exp dislocations/ or exp "fractures, bone"/ or "fractures, cartilage"/ or exp hand injuries/ or exp hip injuries/ or exp leg injuries/ or exp neck injuries/ or occupational injuries/ or soft tissue injuries/ or exp spinal injuries/ or exp "sprains and strains"/ or exp tendon injuries/ or intervertebral disc degeneration/ or intervertebral disc displacement/ or musculoskeletal pain/ or exp back pain/ or Sciatica/ or neck pain/ or myofascial pain syndromes/ or exp tendinopathy/ or patellofemoral pain syndrome/ or tennis elbow/ or fasciitis, plantar/ or heel spur/ or bursitis/ or shoulder impingement syndrome/

17. ((Pain* or ache* or discomfort* or injur* or sore* or excruciat* or tear or tears or injur* or sprain* or strain* or dislocat* or impingement or instabilit* or fracture*) adj8 (musc* or MSK or tendon* or ligament* or joint or joints or bone or bones or soft-tissue or spine or cranial or neck or arm or arms or shoulder* or elbow* or wrist* or hand* or lumbar or back or hip* or knee* or ankle* or foot or feet or heel* or pelvic or rotator cuff or lower extremit* or lower limb* or upper extremit* or upper limb* or leg or legs)).mp.

18. (injur* adj5 (repetitive or overexertion* or lifting or manual handling)).mp.

19. (LBP or lumbago or backache or whiplash or sciatica or carpal tunnel or tendinitis or tendinosis or tendinopath* or axial-pain or spinal injur* or spinal pain or frozen shoulder or shoulder impingement or myofascial pain or patellofemoral pain or regional pain disorder* or cumulative trauma disorder* or osteoarthritis or (hernia* adj3 (disc or discs)) or (injur* adj5 (repetitive or overexertion* or lifting)) or tennis-elbow or epicondylitis or compartment-syndrome or myositis or polymyositis or pyomyositis or bursitis or chondritis or enthesitis or osteitis or epicondylitis or periostitis or periarthritis or synovitis).mp.

20. or/16-19

21. ((predict* or risk or screen*) adj8 (Likert or scale* or questionnaire* or index or checklist* or tool or tools or test or tests or instrument or instruments or score* or inventory)).mp.

22. (job matching or job fit or jobfit or ((pre-employment or job-specific or employment or workhab) adj5 (functional capacity or functional testing or screening or examination*))).mp.

23. (job assessment* or work assessment* or workplace systems assessment* or ergonomic assessment* or (risk adj4 assess* adj8 (job* or work* or ergonomic* or occupation*))).mp.

24. or/21-23

25. (Rapid entire body assessment or Rapid Upper limb assessment or (liberty mutual* adj4 tables) or Snook tables or NIOSH lifting equation* or quick exposure check* or occupational repetitive actions or "Washington Industrial Safety and Health Act" or WISHA or quick ergonomic check or Dutch Musculoskeletal Questionnaire or Assessment of Repetitive Task* or Cumulative Trauma Disorder Risk Assessment Model or Hand Activity Level or ((Threshold Limit value or tlv) adj4 hand activity) or Hand Arm Risk Assessment Method or Keyserling* Cumulative Trauma Checklist or Key Indicator Methods or Loading on the Upper Body Assessment or Ovako Working Posture Analysis System or "Posture, Activity, Tools, and Handling" or PLIBEL or Risk Management Assessment Tool for Manual Handling Proactively or Workplace Ergonomic Risk Assessment or Job strain index or 3DSSPP or senz or life booster or jobfit systems or Pre-Employment Functional Assessments or PEFAs or Industrial Lumbar Motion Monitor or ergoweb or humantech or higher level screening tool).mp.

26. ((QEC or REBA or RULA or OCRA or LUBA or OWAS or PEFA) and (ergonomic* or injur* or risk or job or musculoskeletal or posture or work-related or occupation* or msk or pain)).mp.

27. 25 or 26

28. (15 and 20 and 24) or 27

29. exp Clinical trial/ or (randomi* or randomly or quasi-random* or quasirandom* or groups or subgroups or trial or placebo).tw. or (random adj4 (allocat* or distribut* or assign*)).tw.

30. (((interventional or clinical or experimental) adj1 (design or study or research)) or control group).mp.

31. (("N of 1" or single subject or single case) adj3 (experiment* or design or study or research)).mp.

32. Comparative studies/ or Epidemiologic studies/ or exp case control studies/ or exp cohort studies/ or ((observational adj (study or studies)) or case-control or cohort or follow-up or longitudinal or prospective or Retrospective or consecutive or long-term or longterm or matched-pair or baseline or comparative or pilot study or pilot project).mp.

33. or/29-32

34. 28 and 33

Embase 1974 to 2021 March 18 (OVID Interface)

Date searched: March 19, 2021

Results: 2959

1. (((hospital adj (worker* or employee*)) or healthcare aide* or surgeon* or doctor* or physician* or general practitioner* or nurse* or dentist* or hygienist* or masseur* or massage therapist*) and (pain or safety or safe or unsafe* or accident* or injur* or re-injur* or ergonomic or postur*)).ti.

2. occupational accident/ or workman compensation/ or exp Occupational Health/ or employment/ or Work/ or job performance/ or manual labor/ or shift work/ or work capacity/ or work environment/ or workplace/

3. (exp industry/ or exp nonmedical occupations/) not (athlete/ or military personnel/ or veteran/)

4. (white-collar or blue-collar or shift-worker* or attorney* or lawyer* or miners or mining-industry or office-worker* or desk-worker* or secretarial-worker* or administrative-assistant* or secretaries or manual-labourer* or manual-laborer* or physical-labourer* or physical-laborer* or farmworker* or ((warehous* or construction or farm or agricultur* or fast-food or food-service or grocery or store or retail or maintenance) adj3 (work* or employ* or job* or labor* or labour* or industry)) or steelworker* or iron worker* or ironworker* or mill-worker* or railway-worker* or vehicle-operator* or equipment-operator* or machinery-operator* or transit-operator* or bus-driver* or government-employees or hairdresser* or barber* or esthetician or child-care-providers or child-care-workers or housecleaners or janitor*).mp.

5. (occupation* or (work* not social work) or employ*).jx.

6. ((work* not working-memory) or occupation* or employ* or job or jobs).ti,kw.

7. (work environment* or workplace* or work place* or worksite* or work site* or jobsite or job site or "at work" or "on the job" or "while working" or Work-related or work* compensation).mp.

8. (accident/ or accident prevention/ or accidental injury/ or injury/) and (employ* or (work* not working memory) or occupation* or job* or industr* or labourer* or laborer*).mp.

9. ((safety or safe or safely or unsafe* or accident* or hazard* or injur* or re-injur*) adj10 (employ* or (work* not working-memory) or occupation* or job* or industr* or labourer* or laborer*)).mp.

10. occupational disease*.mp.

11. (job-specific or work-specific or preemployment or pre-employment or work assessment or job assessment or jobfit or job matching).mp.

12. or/1-11

13. musculoskeletal disease/ or ankylosis/ or temporomandibular ankylosis/ or arthropathy/ or ankle instability/ or arthralgia/ or joint contracture/ or joint degeneration/ or joint destruction/ or joint effusion/ or joint laxity/ or joint limitation/ or joint stiffness/ or joint swelling/ or neuropathic joint disease/ or patellofemoral pain syndrome/ or exp periarticular joint disease/ or temporomandibular joint disorder/ or exp osteoarthritis/ or exp joint injury/ or exp joint instability/ or osteoarthropathy/ or exp bone injury/ or fasciitis/ or fascia disease/ or eosinophilic fasciitis/ or plantar fasciitis/ or exp contracture/ or exp enthesopathy/ or exp ligament disease/ or exp limb injury/ or exp limb pain/ or muscle disease/ or anterior tibial syndrome/ or muscle atrophy/ or exp muscle contracture/ or muscle diastasis/ or muscle hypertrophy/ or muscle injury/ or muscle rigidity/ or muscle strain/ or muscle tightness/ or myalgia/ or exp compartment syndrome/ or myositis/ or polymyositis/ or pyomyositis/ or neuromuscular disease/ or musculoskeletal chest pain/ or exp musculoskeletal injury/ or exp musculoskeletal pain/ or exp musculoskeletal stiffness/ or musculoskeletal system inflammation/ or bursitis/ or exp chondritis/ or enthesitis/ or osteitis/ or exp epicondylitis/ or periostitis/ or exp periarthritis/ or exp synovitis/ or exp tendon disease/ or sciatica/ or injury/ or crush trauma/ or limb injury/ or microtrauma/

14. ((Pain* or ache* or discomfort* or injur* or sore* or excruciat* or tear or tears or injur* or sprain* or strain* or dislocat* or impingement or instabilit* or fracture*) adj8 (musc* or MSK or tendon* or ligament* or joint or joints or bone or bones or soft-tissue or spine or cranial or neck or arm or arms or shoulder* or elbow* or wrist* or hand* or lumbar or back or hip* or knee* or ankle* or foot or feet or heel* or pelvic or rotator cuff or lower extremit* or lower limb* or upper extremit* or upper limb* or leg or legs)).mp.

15. (injur* adj5 (repetitive or overexertion* or lifting or manual handling)).mp.

16. (LBP or lumbago or backache or whiplash or sciatica or spinal-pain or spinal-injury or tendinitis or tendinosis or tendinopathy or carpal tunnel or frozen shoulder or shoulder impingement or tennis-elbow or epicondylitis or bursitis or chondritis or enthesitis or osteitis or periostitis or myofascial pain or regional pain disorder* or cumulative trauma disorder* or disc-displacement or disc-degeneration or (hernia* adj3 (disc or discs))).mp.

17. or/13-16

18. ((predict* or risk or screen*) adj8 (Likert or scale* or questionnaire* or index or checklist* or tool or tools or test or tests or instrument or instruments or score* or inventory)).mp.

19. (job matching or job fit or jobfit or ((pre-employment or job-specific or employment or workhab) adj5 (functional capacity or functional testing or screening or examination*))).mp.

20. (job assessment* or work assessment* or workplace systems assessment* or ergonomic assessment* or (risk adj4 assess* adj8 (job* or work* or ergonomic* or occupation*))).mp.

21. or/18-20

22. (Rapid entire body assessment or Rapid Upper limb assessment or (liberty mutual* adj4 tables) or Snook tables or NIOSH lifting equation* or quick exposure check* or occupational repetitive actions or "Washington Industrial Safety and Health Act" or WISHA or quick ergonomic check or Dutch Musculoskeletal Questionnaire or Assessment of Repetitive Task* or Cumulative Trauma Disorder Risk Assessment Model or Hand Activity Level or ((Threshold Limit value or tlv) adj4 hand activity) or Hand Arm Risk Assessment Method or Keyserling* Cumulative Trauma Checklist or Key Indicator Methods or Loading on the Upper Body Assessment or Ovako Working Posture Analysis System or "Posture, Activity, Tools, and Handling" or PLIBEL or Risk Management Assessment Tool for Manual Handling Proactively or Workplace Ergonomic Risk Assessment or Job strain index or 3DSSPP or senz or life booster or jobfit systems or Pre-Employment Functional Assessments or PEFAs or Industrial Lumbar Motion Monitor or ergoweb or humantech or higher level screening tool).mp.

23. ((QEC or REBA or RULA or OCRA or LUBA or OWAS or PEFA) and (ergonomic* or injur* or risk or job or musculoskeletal or posture or work-related or occupation*)).mp.

24. 22 or 23

25. (12 and 17 and 21) or 24

26. limit 25 to conference abstracts

27. 25 not 26

28. exp Clinical trial/ or (randomi* or randomly or quasi-random* or quasirandom* or groups or subgroups or trial or placebo).tw. or (random adj4 (allocat* or distribut* or assign*)).tw.

29. (((interventional or clinical or experimental) adj1 (design or study or research)) or control group).mp.

30. (("N of 1" or single subject or single case) adj3 (experiment* or design or study or research)).mp.

31. Comparative study/ or Clinical study/ or exp Case control study/ or Longitudinal study/ or Retrospective study/ or Prospective study/ or Cohort analysis/ or ((observational adj (study or studies)) or pilot study or pilot project or case-control or

cohort or follow-up or longitudinal or prospective or Retrospective or consecutive or long-term or longterm or baseline or comparative).mp.

32. 28 or 29 or 30 or 31

33. 27 and 32

APA PsycInfo 1806 to November Week 3 2021

Date searched: November 25, 2021

Results: 367

1. occupational health/ or work related illnesses/ or occupational safety/
2. working conditions/ or working space/
3. industrial accidents/
4. workplace intervention/
5. Job Performance/ or Employee Productivity/
6. exp *health personnel/
7. professional personnel/ or librarians/ or engineers/
8. personnel/ or exp artists/ or exp "business and industrial personnel"/ or child care workers/ or exp emergency personnel/ or exp government personnel/
9. occupations/ or job characteristics/
10. hospitality industry/
11. (miners or mining industry or office worker* or desk worker* or secretarial worker* or administrative assistant* or secretaries or manual labourer* or manual laborer* or physical labourer* or physical laborer* or ((warehous* or construction or farm or agricultur* or fast food or food-service or grocery or store or retail) adj3 (work* or employ* or job* or labor* or labour* or industry)) or steelworker* or iron worker* or ironworker* or mill worker* or railway worker* or vehicle operator* or equipment operator* or machinery operator* or transit operator* or bus driver* or government employees or hairdresser* or barber*).mp.
12. (((hospital adj (worker* or employee*)) or healthcare aide* or surgeon* or doctor* or physician* or general practitioner* or nurse* or dentist* or hygienist* or masseur* or masseuse or massage therapist*) and (pain or safety or safe or unsafe* or accident* or injur* or re-injur* or ergonomic or postur*)).ti.

13. (occupation* or (work* not social work) or employ*).jx.

14. ((work* not working-memory) or occupation* or employ* or job or jobs).ti,id.

15. (work-related or job-related or employment-related or work environment* or workplace* or work place* or worksite* or work site* or jobsite or job site or "at work" or "on the job" or "while working" or Work-related or work* compensation).mp.

16. (Accidents/ or Accident Prevention/ or Safety/) and (employ* or work* or occupation* or job* or industr* or labourer* or laborer*).mp.

17. ((safety or safe or safely or unsafe* or accident* or hazard* or injur* or re-injur*) adj10 (employ* or (work* not working-memory) or occupation* or job* or industr* or labourer* or laborer*)).mp.

18. occupational disease*.mp.

19. (job-specific or work-specific or preemployment or pre-employment or work assessment or job assessment or jobfit or job matching).mp.

20. or/1-19

21. exp musculoskeletal disorders/

22. back pain/ or chronic pain/ or myofascial pain/ or exp neuralgia/

23. injuries/

24. ((Pain* or ache* or discomfort* or injur* or sore* or excruciat* or tear or tears or injur* or sprain* or strain* or dislocat* or impingement or instabilit* or fracture*) adj8 (musc* or MSK or tendon* or ligament* or joint or joints or bone or bones or soft-tissue or spine or cranial or neck or arm or arms or shoulder* or elbow* or wrist* or hand* or lumbar or back or hip* or knee* or ankle* or foot or feet or heel* or pelvic or rotator cuff or lower extremit* or lower limb* or upper extremit* or upper limb* or leg or legs)).mp.

25. (injur* adj5 (repetitive or overexertion* or lifting or manual handling)).mp.

26. (LBP or lumbago or backache or whiplash or sciatica or carpal tunnel or tendinitis or tendinosis or tendinopath* or axial-pain or spinal injur* or spinal pain or frozen shoulder or shoulder impingement or myofascial pain or patellofemoral pain or regional pain disorder* or cumulative trauma disorder* or osteoarthritis or (hernia* adj3 (disc or discs)) or (injur* adj5 (repetitive or overexertion* or lifting)) or tennis-elbow or epicondylitis or compartment-syndrome or myositis or polymyositis or pyomyositis or bursitis or chondritis or enthesitis or osteitis or epicondylitis or periostitis or periarthritis or synovitis).mp.

27. or/21-26

28. ((predict* or risk or screen*) adj8 (Likert or scale* or questionnaire* or index or checklist* or tool or tools or test or tests or instrument or instruments or score* or inventory)).mp.

29. (job matching or job fit or jobfit or ((pre-employment or job-specific or employment or workhab) adj5 (functional capacity or functional testing or screening or examination*))).mp.

30. (job assessment* or work assessment* or workplace systems assessment* or ergonomic assessment* or (risk assessment adj8 (job* or work* or ergonomic* or occupation*))).mp.

31. 28 or 29 or 30

32. (Rapid entire body assessment or Rapid Upper limb assessment or (liberty mutual* adj4 tables) or Snook tables or NIOSH lifting equation* or quick exposure check* or occupational repetitive actions or "Washington Industrial Safety and Health Act" or WISHA or quick ergonomic check or Dutch Musculoskeletal Questionnaire or Assessment of Repetitive Task* or Cumulative Trauma Disorder Risk Assessment Model or Hand Activity Level or ((Threshold Limit value or tlv) adj4 hand activity) or Hand Arm Risk Assessment Method or Keyserling* Cumulative Trauma Checklist or Key Indicator Methods or Loading on the Upper Body Assessment or Ovako Working Posture Analysis System or "Posture, Activity, Tools, and Handling" or PLIBEL or Risk Management Assessment Tool for Manual Handling Proactively or Workplace Ergonomic Risk Assessment or Job strain index or 3DSSPP or senz or life booster or jobfit systems or Pre-Employment Functional Assessments or PEFAs or Industrial Lumbar Motion Monitor or ergoweb or humantech or higher level screening tool).mp.

33. ((QEC or REBA or RULA or OCRA or LUBA or OWAS or PEFA) and (ergonomic* or injur* or pain or risk or job or musculoskeletal or msk or pain or posture or work-related or occupation*)).mp.

34. 32 or 33

35. (20 and 27 and 31) or 34

36. exp clinical trials/

37. exp experimental design/

38. (randomi or randomly or quasi-random* or quasirandom* or groups or subgroups or trial or placebo or (random adj4 (allocat* or distribut* or assign*))).tw.

39. (((interventional or clinical or experimental) adj1 (design or study or research)) or control group).mp.

40. (("N of 1" or single subject or single case) adj3 (experiment* or design or study or research)).mp.

41. ((observational adj (study or studies)) or case-control or cohort or follow-up or longitudinal or prospective or Retrospective or consecutive or long-term or longterm or matched pair* or baseline or comparative or pilot study or pilot project).mp.

42. or/36-41

43. 35 and 42

Cochrane Library (Trials database only) (Wiley Interface)

Date searched: March 19, 2021

Results: 2313

#1 [mh ^"Accidents, Occupational"] or [mh ^"workers' compensation"] or [mh ^"Occupational Health"] or [mh ^"Occupational Injuries"] or [mh ^"Occupational Health Services"] or [mh ^"employment"] or [mh ^"Work"] or [mh ^"work performance"] or [mh ^"Workplace"] or [mh "health personnel"[mj]] or or [mh ^"industry"] or [mh ^"beauty culture"] or [mh ^"forestry"] or [mh ^"health care sector"] or [mh ^"laundering"] or [mh ^"manufacturing industry"] or [mh ^"construction industry"] or [mh ^"chemical industry"] or [mh "extraction and processing industry"] or [mh ^"textile industry"] or [mh ^"power plants"] or [mh ^"occupational groups"] or [mh ^"administrative personnel"] or [mh ^"farmers"] or [mh ^"government employees"] or [mh ^"laboratory personnel"] or [mh ^"metal workers"] or [mh ^"miners"]

#2 (white-collar or blue-collar or shift-worker* or attorney* or lawyer* or miners or mining-industry or office-worker* or desk-worker* or secretarial-worker* or administrative-assistant* or secretaries or manual-labourer* or manual-laborer* or physical-labourer* or physical-laborer* or farmworker* or ((warehous* or construction or farm or agricultur* or fast-food or food-service or grocery or store or retail or maintenance) near/3 (work* or employ* or job* or labor* or labour* or industry)) or steelworker* or iron-worker* or ironworker* or mill-worker* or railway-worker* or vehicle-operator* or equipment-operator* or machinery-operator* or transit-operator* or bus-driver* or government-employees or hairdresser* or barber* or esthetician or child-care-providers or child-care-workers or housecleaners or janitor*):ti,ab,kw

#3 (((hospital near/2 (worker* or employee*)) or healthcare-aide or surgeon* or doctor* or physician* or general-practitioner or nurse* or dentist* or hygienist* or masseur* or masseuse or massage-therapist) and (pain or safety or safe or unsafe* or accident* or injur* or re-injur* or ergonomic or postur* or msk or strain or musculoskeletal)):ti

#4 (occupation* or (work* not social work) or employ*):so

#5 ((work* not working-memory) or occupation* or employ* or job or jobs):ti

#6 ([mh ^"Accidents"] or [mh ^"Accident Prevention"] or [mh ^"Safety"]) and (employ* or work* or occupation* or job* or industr* or labourer* or laborer*):ti,ab,kw

#7 ((safety or safe or safely or unsafe* or accident* or hazard* or injur* or re-injur*) near/10 (employ* or (work* not working-memory) or occupation* or job* or industr* or labourer* or laborer*)):ti,ab,kw

#8 (job-specific or work-specific or preemployment or pre-employment or work assessment or job assessment or jobfit or job matching or occupational-disease or occupational-health):ti,ab,kw

#9 #1 OR #2 OR #3 OR #4 OR #5 OR #6 OR #7 OR #8

#10 [mh ^"Musculoskeletal Diseases"] or [mh ^"joint instability"] or [mh ^"joint loose bodies"] or [mh ^"synovitis"] or [mh ^"ischemic contracture"] or [mh ^"contracture"] or [mh ^"dupuytren contracture"] or [mh "arm injuries"] or [mh "back injuries"] or [mh ^"contusions"] or [mh "dislocations"] or [mh "fractures, bone"] or [mh ^"fractures, cartilage"] or [mh "hand injuries"] or [mh "hip injuries"] or [mh "leg injuries"] or [mh "neck injuries"] or [mh ^"occupational injuries"] or [mh ^"soft tissue injuries"] or [mh "spinal injuries"] or [mh "sprains and strains"] or [mh "tendon injuries"] or [mh ^"intervertebral disc degeneration"] or [mh ^"intervertebral disc displacement"] or [mh ^"musculoskeletal pain"] or [mh "back pain"] or [mh ^"Sciatica"] or [mh ^"neck pain"] or [mh ^"myofascial pain syndromes"] or [mh "tendinopathy"] or [mh ^"patellofemoral pain syndrome"] or [mh ^"tennis elbow"] or [mh ^"fasciitis, plantar"] or [mh ^"heel spur"] or [mh ^"bursitis"] or [mh ^"shoulder impingement syndrome"]

#11 ((Pain* or ache* or discomfort* or injur* or sore* or excruciat* or tear or tears or injur* or sprain* or strain* or dislocat* or impingement or instabilit* or fracture*) near/8 (musc* or MSK or tendon* or ligament* or joint or joints or bone or bones or soft-tissue or spine or cranial or neck or arm or arms or shoulder* or elbow* or wrist* or hand* or lumbar or back or hip* or knee* or ankle* or foot or feet or heel* or pelvic or rotator-cuff or lower-extremities or lower-limb or upper-extremities or upper-limb or leg or legs)):ti,ab,kw

#12 (injur* near/5 (repetitive or overexertion* or lifting or manual handling)):ti,ab,kw

#13 (LBP or lumbago or backache or whiplash or sciatica or carpal tunnel or tendinitis or tendinosis or tendinopath* or axial-pain or spinal-injur* or spinal-pain or frozen-shoulder or shoulder-impingement or myofascial-pain or patellofemoral-pain or regional-pain-disorder* or cumulative-trauma-disorder* or osteoarthritis or (hernia* near/3 (disc or discs)) or (injur* near/5 (repetitive or overexertion* or lifting)) or tennis-elbow or epicondylitis or compartment-syndrome or myositis or polymyositis or pyomyositis or

bursitis or chondritis or enthesitis or osteitis or epicondylitis or periostitis or periarthritis or synovitis):ti,ab,kw

#14 #10 OR #11 OR #12 OR #13

#15 ((predict* or risk or screen*) near/8 (Likert or scale* or questionnaire* or index or checklist* or tool or tools or test or tests or instrument or instruments or score* or inventory)):ti,ab,kw

#16 (job-matching or job-fit or jobfit or ((pre-employment or job-specific or employment or workhab) near/5 (functional-capacity or functional-testing or screening or examination*))):ti,ab,kw

#17 (job assessment* or work assessment* or workplace systems assessment* or ergonomic assessment* or (risk near/4 assess* near/8 (job* or work* or ergonomic* or occupation*))):ti,ab,kw

#18 #15 OR #16 OR #17

#19 ("Rapid entire body assessment" or "Rapid Upper limb assessment" or (liberty-mutual near/4 tables) or Snook-tables or NIOSH-lifting-equation or quick-exposure-check or occupational-repetitive-actions or "Washington Industrial Safety and Health Act" or WISHA or quick-ergonomic-check or Dutch-Musculoskeletal-Questionnaire or Assessment-of-Repetitive-Task* or Cumulative-Trauma-Disorder-Risk-Assessment-Model or Hand-Activity-Level or ((Threshold-Limit-value or tlv) near/4 hand-activity) or Hand-Arm-Risk-Assessment-Method or Keyserling-Cumulative-Trauma-Checklist or Key-Indicator-Methods or Loading-on-the-Upper-Body-Assessment or Ovako-Working-Posture-Analysis-System or "Posture, Activity, Tools, and Handling" or PLIBEL or "Risk Management Assessment Tool for Manual Handling Proactively" or "Workplace Ergonomic Risk Assessment" or "Job strain index" or 3DSSPP or senz or life booster or jobfit systems or "Pre-Employment Functional Assessments" or PEFAs or "Industrial Lumbar Motion Monitor" or ergoweb or humantech or "higher level screening tool"):ti,ab,kw

#20 ((QEC or REBA or RULA or OCRA or LUBA or OWAS or PEFA) and (ergonomic* or injur* or risk or job or musculoskeletal or posture or work-related or occupation* or msk or pain)):ti,ab,kw

#21 #19 OR #20

#22 (#9 AND #14 AND #18) OR #21

CINAHL Plus with Full Text (Ebscohost interface)

Date searched: March 19, 2021

Results: 1686

Deselect "Apply equivalent subjects" to all search lines

S1 (miners or mining industry or office worker* or desk worker* or secretarial worker* or administrative assistant* or secretaries or manual labourer* or manual laborer* or physical labourer* or physical laborer* or ((warehous* or construction or farm or agricultur* or fast food or food-service or grocery or store or retail) N3 (work* or employ* or job* or labor* or labour* or industry)) or steelworker* or iron worker* or ironworker* or mill worker* or railway worker* or vehicle operator* or equipment operator* or machinery operator* or transit operator* or bus driver* or government employees or hairdresser* or barber*)

S2 TI(((hospital N1 (worker* or employee*)) or healthcare aide* or surgeon* or doctor* or physician* or general practitioner* or nurse* or dentist* or hygienist* or masseur* or masseuse or massage therapist*) and (pain or safety or safe or unsafe* or accident* or injur* or re-injur* or ergonomic or postur* or msk or strain or musculoskeletal))

S3 SO(occupation* or (work* not social work) or employ*)

S4 TI((work* not working-memory) or occupation* or employ* or job or jobs)

S5 (work-related or job-related or employment-related or working-conditions or work-environment* or workplace* or work-place* or worksite* or work-site* or jobsite or job-site or "at work" or "on the job" or "while working" or work* compensation or occupational-disease* or job-specific or work-specific or preemployment or pre-employment or work-assessment or job-assessment or jobfit or job-matching)

S6 ((MH "Safety") OR (MH "Accidents")) and (employ* or work* or occupation* or job* or industr* or labourer* or laborer*)

S7 ((safety or safe or safely or unsafe* or accident* or hazard* or injur* or re-injur*) N10 (employ* or (work* not working-memory) or occupation* or job* or industr* or labourer* or laborer*))

S8 (MH "Accidents, Occupational+") OR (MH "Occupational Health+") OR (MH "Impairment, Health Professional") or (MH "Work Environment") OR (MH "Employment") OR (MH "Occupations and Professions") OR (MH "Worker's Compensation") OR (MH "Named Groups by Occupation") OR (MH "Blue Collar Workers") OR (MH "Child Care Providers") OR (MH "Correctional Facilities Personnel") OR (MH "Farmworkers") OR (MH "Firefighters") OR (MH "Government Employees") OR (MM "Health Personnel+") OR (MH "Librarians+") OR (MH "Pilots") OR (MH "Teachers") OR (MH "White Collar Workers") OR (MH "Administrative Personnel") OR (MH "Attorneys+") OR (MH "Estheticians")

S9 S1 OR S2 OR S3 OR S4 OR S5 OR S6 OR S7 OR S8

S10 (MH "Musculoskeletal Diseases+") OR (MH "Facial Pain") OR (MH "Knee Pain+") OR (MH "Muscle Pain") OR (MH "Referred Pain") OR (MH "Neck Pain") OR (MH "Neuralgia+") or (MH "Leg Injuries+") OR (MH "Ligament Injuries+") OR (MH "Neck Injuries+") OR (MH "Sprains and Strains+") OR (MH "Tendon Injuries+") OR (MH "Hand Injuries+") OR (MH "Dislocations+") OR (MH "Back Injuries+") OR (MH "Arm Injuries+") OR (MH "Accidental Injuries") OR (MH "Crush Injuries") OR (MH "Fractures+") OR (MH "Occupational-Related Injuries") OR (MH "Soft Tissue Injuries+") OR (MH "Spinal Injuries+") OR (MH "Tears and Lacerations+")

S11 ((Pain* or ache* or discomfort* or injur* or sore* or excruciat* or tear or tears or injur* or sprain* or strain* or dislocat* or impingement or instabilit* or fracture*) N8 (musc* or MSK or tendon* or ligament* or joint or joints or bone or bones or soft-tissue or spine or cranial or neck or arm or arms or shoulder* or elbow* or wrist* or hand* or lumbar or back or hip* or knee* or ankle* or foot or feet or heel* or pelvic or rotator-cuff or lower-extremit* or lower-limb* or upper-extremit* or upper-limb* or leg or legs)) OR (injur* N5 (repetitive or overexertion* or lifting or manual handling)) OR LBP or lumbago or backache or whiplash or sciatica or carpal-tunnel or tendinitis or tendinosis or tendinopath* or axial-pain or spinal-injur* or spinal-pain or frozen-shoulder or shoulder-impingement or myofascial-pain or patellofemoral-pain or regional-pain-disorder* or cumulative-trauma-disorder* or osteoarthritis or (hernia* N3 (disc or discs)) or (injur* N5 (repetitive or overexertion* or lifting)) or tennis-elbow or epicondylitis or compartment-syndrome or myositis or polymyositis or pyomyositis or bursitis or chondritis or enthesitis or osteitis or epicondylitis or periostitis or periarthritis or synovitis

S12 ((predict* or risk or screen*) N8 (Likert or scale* or questionnaire* or index or checklist* or tool or tools or test or tests or instrument or instruments or score* or inventory)) OR job matching or job fit or jobfit or ((pre-employment or job-specific or employment or workhab) N5 (functional capacity or functional testing or screening or examination*)) OR job-assessment* or work-assessment* or workplace-systems-assessment* or ergonomic-assessment* or ((risk N4 assess*) N8 (job* or work* or ergonomic* or occupation*))

S13 (Rapid-entire-body-assessment or Rapid-Upper-limb-assessment or (liberty-mutual* N4 tables) or Snook-tables or NIOSH-lifting-equation* or quick-exposure-check* or occupational-repetitive-actions or "Washington Industrial Safety and Health Act" or WISHA or quick-ergonomic-check or Dutch-Musculoskeletal-Questionnaire or Assessment-of-Repetitive-Task* or Cumulative-Trauma-Disorder-Risk-Assessment-Model or Hand-Activity-Level or ((Threshold-Limit-value or tlv) N4 hand-activity) or Hand-Arm-Risk-Assessment-Method or Keyserling*-Cumulative-Trauma-Checklist or Key-Indicator-Methods or Loading-on-the-Upper-Body-Assessment or Ovako-Working-

Posture-Analysis-System or "Posture, Activity, Tools, and Handling" or PLIBEL or Risk-Management-Assessment-Tool for Manual-Handling-Proactively or Workplace-Ergonomic-Risk-Assessment or Job-strain-index or 3DSSPP or senz or life-booster or jobfit-systems or Pre-Employment-Functional-Assessments or PEFAs or Industrial-Lumbar-Motion-Monitor or ergoweb or humantech or higher-level-screening-tool) OR ((QEC or REBA or RULA or OCRA or LUBA or OWAS or PEFA) and (ergonomic* or injur* or risk or job or musculoskeletal or posture or work-related or occupation* or msk or pain))

S14 (S9 AND (S10 OR S11) AND S12) OR S13

S15 ((MH "Experimental Studies") OR (MH "Clinical Trials+") OR (MH "Community Trials") OR (MH "Controlled Before-After Studies") OR (MH "Nonrandomized Trials") OR (MH "Static Group Comparison") OR (MH "Pretest-Posttest Design+")) OR ((randomi* or randomly or quasi-random* or quasirandom* or groups or subgroups or trial or placebo) or (random N4 (allocat* or distribut* or assign*))) OR ((((interventional or clinical or experimental) N1 (design or study or research)) or control group)) OR ((("N of 1" or single subject or single case) N3 (experiment* or design or study or research)))

S16 ((MH "Case Control Studies+") OR (MH "Double-Blind Studies") OR (MH "Prospective Studies+") OR (MH "Single-Blind Studies") OR (MH "Triple-Blind Studies")) OR (((observational N1 (study or studies)) or case-control or cohort or follow-up or longitudinal or prospective or Retrospective or consecutive or long-term or longterm or matched-pair or baseline or comparative or pilot study or pilot project))

S17 S15 OR S16

S18 S14 AND S17

Scopus

Date searched: March 19, 2021

Results: 3173

#1 TITLE-ABS-KEY (white-collar OR blue-collar OR shift-worker* OR attorney* OR lawyer* OR miners OR mining-industry OR office-worker* OR desk-worker* OR secretarial-worker* OR administrative-assistant* OR secretaries OR manual-labourer* OR manual-laborer* OR physical-labourer* OR physical-laborer* OR farmworker* OR ((warehous* OR construction OR farm OR agricultur* OR fast-food OR food-service OR grocery OR store OR retail OR maintenance) W/3 (work* OR employ* OR job* OR labor* OR labour* OR industry)) OR steelworker* OR iron-worker* OR ironworker* OR mill-worker* OR railway-worker*

OR vehicle-operator* OR equipment-operator* OR machinery-operator* OR transit-operator* OR bus-driver* OR government-employees OR hairdresser* OR barber* OR esthetician OR child-care-providers OR child-care-workers OR housecleaners OR janitor*) OR TITLE (((hospital W/1 (worker* OR employee*)) OR healthcare-aide* OR surgeon* OR doctor* OR physician* OR general-practitioner* OR nurse* OR dentist* OR hygienist* OR masseur* OR masseuse OR massage-therapist*) AND (pain OR safety OR safe OR unsafe* OR accident* OR injur* OR re-injur* OR ergonomic OR postur* OR msk OR strain OR musculoskeletal)) OR SRCTITLE (occupation* OR (work* AND NOT social-work) OR employ*) OR TITLE ((work* AND NOT working-memory) OR occupation* OR employ* OR job OR jobs) OR TITLE-ABS-KEY (work-related OR job-related OR employment-related OR working-conditions OR work-environment* OR workplace* OR work-place* OR worksite* OR work-site* OR jobsite OR job-site OR "at work" OR "on the job" OR "while working" OR work*-compensation OR occupational-disease* OR occupational-health OR occupational-safety OR job-specific OR work-specific OR preemployment OR pre-employment OR work-assessment OR job-assessment OR jobfit OR job-matching) OR (KEY (safety OR accident) AND TITLE-ABS-KEY (employ* OR work* OR occupation* OR job* OR industr* OR labourer* OR laborer*)) OR TITLE-ABS-KEY ((safety OR safe OR safely OR unsafe* OR accident* OR hazard* OR injur* OR re-injur*) W/10 (employ* OR (work* AND NOT working-memory) OR occupation* OR job* OR industr* OR labourer* OR laborer*))

#2 KEY (musculoskeletal-disease OR ankylosis OR temporomandibular-ankylosis OR arthropathy OR ankle-instability OR arthralgia OR joint-contracture OR joint-degeneration OR joint-destruction OR joint-effusion OR joint-laxity OR joint-limitation OR joint-stiffness OR joint-swelling OR neuropathic-joint-disease OR patellofemoral-pain OR periarticular-joint-disease OR temporomandibular-joint-disorder OR osteoarthritis OR joint-injury OR joint-instability OR osteoarthropathy OR bone-injury OR fasciitis OR fascia-disease OR eosinophilic-fasciitis OR plantar-fasciitis OR contracture OR enthesopathy OR ligament-disease OR limb-injury OR limb-pain OR muscle-disease OR anterior-tibial-syndrome OR muscle-atrophy OR muscle-contracture OR muscle-diastasis OR muscle-hypertrophy OR muscle-injury OR muscle-rigidity OR muscle-strain OR muscle-tightness OR myalgia OR compartment-syndrome OR myositis OR polymyositis OR pyomyositis OR neuromuscular-disease OR musculoskeletal-chest-pain OR musculoskeletal-injury OR musculoskeletal-pain OR musculoskeletal-stiffness OR musculoskeletal-system-inflammation OR bursitis OR chondritis OR enthesitis OR osteitis OR epicondylitis OR periostitis OR periarthritis OR synovitis OR tendon-disease OR sciatica OR injury OR crush-trauma OR limb-injury OR microtrauma) OR TITLE-ABS-KEY (((pain* OR ache* OR discomfort* OR injur* OR sore* OR excruciat* OR tear OR tears OR injur* OR sprain* OR strain* OR dislocat* OR

impingement OR instabilit* OR fracture*) W/8 (musc* OR msk OR tendon* OR ligament* OR joint OR joints OR bone OR bones OR soft-tissue OR spine OR cranial OR neck OR arm OR arms OR shoulder* OR elbow* OR wrist* OR hand* OR lumbar OR back OR hip* OR knee* OR ankle* OR foot OR feet OR heel* OR pelvic OR rotator-cuff OR lower-extremit* OR lower-limb* OR upper-extremit* OR upper-limb* OR leg OR legs)) OR (injur* W/5 (repetitive OR overexertion* OR lifting OR manual-handling)) OR lbp OR lumbago OR backache OR whiplash OR sciatica OR carpal-tunnel OR tendinitis OR tendinosis OR tendinopath* OR axial-pain OR spinal-injur* OR spinal-pain OR frozen-shoulder OR shoulder-impingement OR myofascial-pain OR patellofemoral-pain OR regional-pain-disorder* OR cumulative-trauma-disorder* OR osteoarthritis OR (hernia* W/3 (disc OR discs)) OR (injur* W/5 (repetitive OR overexertion* OR lifting)) OR tennis-elbow OR epicondylitis OR compartment-syndrome OR myositis OR polymyositis OR pyomyositis OR bursitis OR chondritis OR enthesitis OR osteitis OR epicondylitis OR periostitis OR periarthritis OR synovitis)

#3 TITLE-ABS-KEY(((predict* or risk or screen*) W/8 (Likert or scale* or questionnaire* or index or checklist* or tool or tools or test or tests or instrument or instruments or score* or inventory)) OR job-matching or job-fit or jobfit or ((pre-employment or job-specific or employment or workhab) W/5 (functional-capacity or functional-testing or screening or examination*)) OR job-assessment* or work-assessment* or workplace-systems-assessment* or ergonomic-assessment* or ((risk W/4 assess*) W/8 (job* or work* or ergonomic* or occupation*)))

#4 TITLE-ABS-KEY(Rapid-entire-body-assessment or Rapid-Upper-limb-assessment or (liberty-mutual* W/4 tables) or Snook-tables or NIOSH-lifting-equation* or quick-exposure-check* or occupational-repetitive-actions or "Washington Industrial Safety and Health Act" or WISHA or quick-ergonomic-check or Dutch-Musculoskeletal-Questionnaire or Assessment-of-Repetitive-Task* or Cumulative-Trauma-Disorder-Risk-Assessment-Model or Hand-Activity-Level or ((Threshold-Limit-value or tlv) W/4 hand-activity) or Hand-Arm-Risk-Assessment-Method or Keyserling*-Cumulative-Trauma-Checklist or Key-Indicator-Methods or Loading-on-the-Upper-Body-Assessment or Ovako-Working-Posture-Analysis-System or "Posture, Activity, Tools, and Handling" or PLIBEL or Risk-Management-Assessment-Tool for Manual-Handling-Proactively or Workplace-Ergonomic-Risk-Assessment or Job-strain-index or 3DSSPP or senz or life-booster or jobfit-systems or Pre-Employment-Functional-Assessments or PEFAs or Industrial-Lumbar-Motion-Monitor or ergoweb or humantech or higher-level-screening-tool OR ((QEC or REBA or RULA or OCRA or LUBA or OWAS or PEFA) and (ergonomic* or injur* or risk or job or musculoskeletal or posture or work-related or occupation* or msk or pain)))

#5 (#1 AND #2 AND #3) OR #4

#6 TITLE-ABS-KEY (rct OR randomi* OR randomly OR quasi-random* OR quasirandom* OR groups OR subgroups OR {trial} OR placebo OR (random W/4 (allocat* OR distribut* OR assign*))) OR TITLE-ABS-KEY (((interventional OR clinical OR experimental) W/1 (design OR study OR research)) OR control-group) OR TITLE-ABS-KEY (("N of 1" OR single-subject OR single-case) W/3 (experiment* OR design OR study OR research)) OR KEY (comparative-study OR clinical-study OR case-control-study OR longitudinal-study OR retrospective-study OR prospective-study OR cohort-analysis) OR TITLE-ABS-KEY ((observational W/1 (study OR studies)) OR pilot-study OR pilot-project OR case-control OR cohort OR follow-up OR longitudinal OR prospective OR retrospective OR consecutive OR long-term OR longterm OR baseline OR comparative)

#7 #5 AND #6

Supplementary Material S2: Downs and Black study quality assessment scoring for eligible articles, prior to removal due to poor quality

			Single-intervention included articles					Multiple-intervention included articles							Poor quality articles					
		Possible Score	Frost 2007 [21]	Ho 2014 [22]	Ketola 2002 [23]	Speklé 2010 (RCT) [9]	Speklé 2010 (Economic) [24]	Carrivick 2002 (JOH) [26]	Carrivick 2002 (JOEM) [27]	Carrivick 2005 [28]	Craib 2007 [30]	Porru 2017 [36]	Rautianen 2004 [37]	Oude Hengel 2013 [35]	Carrivick 2001 [25]	Cheng 2009 [29]	Johnson 2002 [31]	Laing 2005 [32]	Laing 2007 [33]	Melhorn 2001 [34]
Reporting	Q1	1	1	1	1	1	1	1	1	1	1	1	1	1	0	1	1	1	1	1
	Q2	1	1	1	1	1	1	1	1	1	1	1	1	1	1	0	0	1	1	1
	Q3	1	1	1	1	1	1	1	1	1	1	0	0	1	1	1	0	0	0	0
	Q4	1	1	1	1	1	1	1	1	1	1	1	0	0	1	1	0	1	1	0
	Q5	2	1	0	1	0	1	0	0	0	0	0	2	1	0	0	0	0	0	1
	Q6	1	1	1	1	1	1	1	1	1	1	1	1	1	1	0	0	1	1	1
	Q7	1	1	1	1	1	1	1	1	1	1	1	0	1	0	1	0	1	1	0
	Q8	1	0	0	0	0	1	0	0	0	0	0	0	0	0	0	0	0	0	0
	Q9	1	0	1	0	1	0	0	0	0	0	0	0	1	0	0	0	0	0	0
	Q10	1	1	1	1	1	1	1	1	1	1	1	1	1	1	1	0	1	1	1
External validity	Q11	1	0	0	0	0	0	1	1	1	0	1	1	1	1	1	0	0	0	0
	Q12	1	0	0	0	0	0	1	1	0	0	1	0	0	1	0	0	0	0	0
	Q13	1	1	1	1	1	1	1	1	1	1	1	1	1	1	1	1	1	1	1
Internal validity (Bias)	Q14	1	0	0	0	0	0	0	0	0	0	0	0	0	0	0	0	0	0	0
	Q15	1	0	0	1	0	0	0	0	0	0	0	0	0	0	0	0	0	0	0
	Q16	1	1	1	1	1	1	1	0	0	1	1	1	1	1	1	0	1	1	1
	Q17	1	1	1	1	1	1	1	1	1	1	1	1	1	0	1	0	1	1	1
	Q18	1	1	1	1	1	1	1	1	1	1	1	1	1	0	1	0	1	1	0
	Q19	1	1	1	1	0	0	1	1	1	0	1	1	0	1	1	0	1	1	1
	Q20	1	0	0	0	1	1	0	0	0	1	1	0	1	0	0	0	0	0	0
Internal validity (confounding)	Q21	1	1	1	1	1	1	1	1	1	0	1	1	1	1	1	0	1	1	0
	Q22	1	1	1	1	1	1	1	1	1	1	1	0	1	1	1	0	1	1	0
	Q23	1	1	1	1	1	1	0	0	0	0	0	1	1	0	1	0	0	0	0
	Q24	1	0	0	0	0	0	0	0	0	0	0	0	0	0	0	0	0	0	0
	Q25	1	0	0	1	1	0	1	1	1	1	0	0	1	1	0	0	0	0	0
	Q26	1	1	1	1	0	0	0	0	0	1	1	0	1	1	0	0	0	0	1
Power	Q27	1	1	1	0	1	1	0	0	0	0	0	1	0	0	0	0	0	0	0
	Total	28	18	18	19	18	18	17	16	15	15	17	15	19	14	14	2	13	13	10

Supplementary Material S3: Characteristics of included studies. Characteristics included design, setting, description of MSI screening tool, study arm description, outcome measurements, and results

Study reference	Study design characteristics	Setting and participant characteristics	Name and Description of MSI Risk Factor Screening Tool(s)	Outcome measures	Study arm descriptions	Description of tool-dependent programmatic intervention	Data source(s) used for assessment of outcomes	Key results
**Single-Tool Included Studies**								
Frost 2007 [21]	Cluster RCT; outcome-specific 6 to 8 quarters follow-up	General working population in Western Denmark: work sites with >20 employees and affiliated to 1 of 3 occupational health centres; 4006 participants randomized; convenience sampling (39 worksites from 293 contacted)	"Danish working environment regulations": assessment of lifting burdens, wheeled equipment use, repetitive risky movement patterns	Work absence:<br>1) >7 day accumulated work absence due to pain (6 quarter follow-up)<br>2) >14 day accumulated work absence from any cause (8 quarter follow-up)	Intervention 1 :<br>Booklet on negative belief patterns and pain; presentation on site-specific workloads (randomized n=1516)<br>Intervention 2:<br>Intervention 1 + application of "Danish working environment regulations" to identify unacceptable heavy lifting, unsafe worksite, awkard posture (randomized n=1374)<br>Control:	Resultant from Intervention 2:<br>When unsafe work site / working conditions identified, further quantification of exposure, and "technical or organizational solutions to eliminate such loads were suggested"	Work absence:<br>Company-provided electronic absence data, provided quarterly	Work absence:<br>No statistically significant difference in HR for pain-related or general absence-taking between any arms<br><br>Among participants with new pain-related absence and employed at follow-up, no statistically significant difference in HR for likelihood of being at work between any arms<br><br>Among participants with new general absence and employed at follow-up, decreased likelihood of booklet+presentation arm being at work (HR 0.80 (95% CI 0.68 - 0.95)); no significant difference for risk

Ho 2014 [22]	Individual RCT; 2 week follow-up	*Trial 1:* Participants from tertiary education institution in Hong Kong; 111 participants randomized; convienence sampling<br><br>*Trial 2:* sedentary worker participants from major banking corportion in Hong Kong; 75 participants randomized; convenience sampling	Display Screen Equipment Risk Assessment and Management System (DSE RAM System): identification of workstation hazards using age, layout, misfit indices, worker preference of monitor and keyboard position; presentation of workstation change-based recommendations	Musculoskeletal discomfort:<br>1) average self-report score pre- and post-intervention (range 0-10; lower= less discomfort) for 9 body parts (trial 1) / 5 body parts (trial 2)<br>2) average combined total self report score pre- and post-intervention (range 0-50; lower = less discomfort) (trial 2)<br><br>Behavioural changes:<br><br>Odds of answering "Yes", acknowleging specific behaviours in previous two weeks	*Both trials:*<br>Intervention: Use of DSE RAM System risk-assessment and immediate recommendation for workstation modification (Trial 1 n=56, Trial 2 n=38)<br>Control: Use of DSE Ram System, recommendations withheld until after follow-up period (Trial 1 n=55; Trial 2 n=37)	Resultant from Intervention:<br>Based on DSE RAM assessment, recommendations to modify workstation provided: included adjustment of display screen height, seat height, desk setup, distance to display screen	Musculoskeletal discomfort: Self-report musculoskeletal discomfort questionnaire<br><br>Self-report computer-specific ocupational health attitudes and behavioural checklist	Musculoskeletal discomfort:<br>Format: pre-intervention -> post-intervention measurements in immediate/delayed intervention group; interaction term present for significance<br>Trial 1: larger pre-post decrease in immediate intervention group for shoulders, elbows, wrists, upper back.<br>*Shoulders:*<br>Immediate intervention group mean(SD): 4.91(2.82) -> 3.78(2.59)<br>Delayed intervention group mean(SD): 5.41(2.67) -> 5.43(2.81); interaction p < 0.05<br>*Elbows*:<br>Immediate intervention group mean(SD): 3.56(2.50) -> 2.62(2.23)<br>Delayed intervention group mean(SD): 3.91(2.65) -> 3.98(2.44); interaction p < 0.05<br>*Wrists:*<br>Immediate intervention group mean(SD): 3.80(2.71) -> 2.60(2.21)<br>Delayed intervention group mean(SD): 4.02(2.65) ->
Ketola 2002 [23]	Individual RCT; follow-up at 2 and 10 months	Office computer users in Finland (more than 4 hours/week using video display units): from 3 administrational units of a medium-sized Finnish city; 124 participants randomized; sampling method unclear	"Ergonomic checklist for VDU work":checklist for workroom layour, workstation adjustments, work breaks	Musculoskeletal discomfort:<br>1) average daily diary self report score at 2- and 10-month follow up (range 1-5; lower=less discomfort) for 18 anatomical areas<br>2) Preceding 30 days musculoskeletal strain self-report score (range 1-5; lower=less strain)<br>3) Preceding 30 days musculoskeletal pain (≥1 day of pain / 0 days)	Intervention 1 (reference): one-page leaflet on VDU work; ergonomic consultation at participant request (n=33)<br>Intervention 2:<br>Intervention 1 + "ergonomic checklist for VDU work", followed by consultation with physiotherapist based on checklist results (n=39)<br>Intervention 3:<br>Intervention 1 + 1-hour ergonomics training session + "ergonomic checklist for VDU work" (n=35)	Resultant from Interventions 2, 3:<br>In response to ergonomic checklist, redesign/rearrangement of workstation, postural recommendations	Musculoskeletal discomfort:<br>2-week daily diary (3 daily measurements) using modified Nordic questionnaire<br><br>Questionnaire on musculoskeletal strain and pain in the preceeding 30 days	Musculoskeletal discomfort:<br>Daily diary scores:<br>Format: group mean score(SE), p-value from Dunnett's test comparing to reference group<br>*2 months:*<br>Neck: Intensive 2.7(0.2), p-value < 0.05; education 2.7(0.1), p < 0.05; reference 3.3(0.2)<br>Area between neck and shoulder (right): intensive 2.5(0.1), p < 0.01; education 2.5(0.1), p < 0.01; reference 3.1(0.2)<br>Right shoulder: intensive 2.2(0.2), p < 0.05; reference 2.8(0.2)<br>Left shoulder: intensive 1.9(0.1), p < 0.05; reference group 2.4(0.2)<br>Left hand fingers: intensive 1.8(0.1), p < 0.05; reference group 2.3(0.1)<br>Upper back: intensive 2.2(0.1), p < 0.01, education 2.4(0.1), p < 0.01, reference 2.9(0.1)<br>*10 months:*<br>NS for any body part<br><br>Questionnaire on musculoskeletal strain and pain (past 30 days):

Speklé 2010 (RCT) [9]	Cluster RCT; 12 month follow-up	Mixed general working population from the Netherlands: 7 Dutch organizations of varying occupational characteristics; 1183 participants randomized; purposive non-random sampling	Repetitive Strain Injury (RSI) QuickScan: questionnaire on exposure to risk factors from occupation, relationship with colleagues, ergonomics and musculoskeletal health	<u>Musculoskeletal discomfort:</u> Prevalence of arm, shoulder, neck symptoms within last 6 months / 7 days; two categories - proximal and distal appendage symptoms (operationalized as prevalence odds)<br><br><u>Work absence:</u> Days of sick leave from study entrance	<u>Intervention 1:</u> Participants completed and immediately received feedback from RSI QuickScan, defining one of 16 interventions (n=365)<br><u>Intervention 2 (control):</u> Participants completed RSI QuickScan, but received only general advice. Individuals reporting severe arm, shoulder, neck symptoms referred to occupational [illegible]	<u>Resultant from Intervention 1:</u> included, but not limited to, change to workstation setup, eyesight assessment, initiation of occupational health physician appointment, shoulder and neck symptom prevention education program, workplace stress training	<u>Musculoskeletal discomfort:</u> Online questionnaire (Modified Nordic questionnaire - 7 regions) of 6-month and 7-day prevalence of arm, shoulder, neck symptoms<br><br><u>Work absence:</u> Sick leave data from human resources department of organizations participating in the study	<u>Musculoskeletal discomfort:</u><br>*12 months:*<br>NS for prevalence of arm, shoulder neck symptoms (total) (95% CI 0.61 - 1.30)<br>NS for prevalence of proximal symptoms (95% CI 0.54 - 1.12)<br>NS for prevalence of distal symptoms (95% CI 0.49 - 1.67)<br><br><u>Work absence:</u><br>*12 months:*<br>NS for days of sick leave
Speklé 2010 (Economic analysis) [24]	see above	see above	see above	<u>Resource utilization:</u> Direct (practitioner) healthcare costs (Euro)<br>Direct non-healthcare costs (Euro)<br>Intervention cost (Euro)<br><br><u>Work absence:</u> Sick leave cost (Euro)	see above	see above	<u>Resource utilization:</u> Online questionnaire (additional to standard RSI QuickScan) to measure resource utilization	<u>Resource utilization:</u><br>*12 months:*<br>Direct (practitioner) healthcare costs: NS between groups<br>Direct non-healthcare costs: NS between groups<br>Intervention cost: mean intervention additional cost of 30.73 (95% CI 18.78 - 41.03) Euro<br>Total direct costs (sum of all direct and intervention costs): NS between groups<br><br>[illegible]

Multiple Tool/Intervention Included Studies

Carrivick 2002 (JOEM) [27]	Quasi-experimental pre-post design with control group; analysis period of 88 months with follow-up associated with duration of employment status	Cleaners (n=137) and orderlies (n=128) from a teaching hospital in Western Australia; nonrandomized	Manual Handling Checklist: assessment of actions, workstataion, posture, qualities of manual handling, loads moved, work environment, skills and demographic factors	Workplace-associated MSI:<br>Pre-post lost time injury count and rate (per hours worked) Frequency distributrion of injury count (0, 1, >1)<br>Work absence:<br>Pre-post working hours lost from injury - count and rate (per hours worked)<br>Claims cost:<br>Pre-post total compensation costs from lost time injuries (count and rate)	Intervention (cleaners) group:<br>52 months pre-intervention period compared to 36-month intervention period. Over course of intervention period, use of Manual Handling Checklist, injury data, communication from staff; eventual categorization of injury risk for specific identified hazards, then providing risk reduction via varied hazard controls (n=137)<br>Control (orderlies) group:<br>52 months pre-intervention period compared to 36-month intervention period. Usual training, access to hospital occupational safety services(n=128)	Resultant from Intervention 1:<br>changes to hospital purchasing criteria, reducing frequency of risky handling procedures, reduction of workplace equipment weight, reduction of frequency of repetitive tasks, improved employee training on safe manual handling	Workplace-associated MSI:<br>Personnel records and incident datasheets<br>Work absence:<br>Personnel records<br>Claims cost:<br>Personnel records, financial records, insurer workers compensation documents	Workplace-associated MSI:<br>Injury frequency distribution: significant pre-post association between proportion of injured (1+ injuries) and non-injured (0 injury) individuals (p < 0.01)<br>*Univariate analysis*<br>**Pre-WRAT mean(SE) -> post-WRAT mean (SE); 95% CI for difference**<br>Workplace-associated MSI:<br>Injury frequency rate (units not given):<br>Cleaners: 0.892(0.148) -> 0.399(0.125); -0.863 - -0.124<br>Orderlies: 1.048(0.158) -> 1.450(0.197); 0.022 - 0.784<br>Work absence:<br>Injury duration rate (units not given):<br>Cleaners: 2.419(0.186) -> 1.765(0.163); -1.069 - -0.239<br>Orderlies: 2.273(0.210) -> 3.112(0.237); 0.334 - 1.344<br>Claims cost:<br>Injury claims cost rate (units not given):<br>Cleaners: 3.285(0.286) -> 2.058(0.228); -1.855 - -0.599<br>Orderlies: 3.263(0.323) -> 4.364(0.365); 0.211 - 1.791<br>*Multivariate (generalized linear mixed modelling) analysis (Odds ratio and*
Carrivick 2002 (JOH) [26]	see above (additional analysis)	see above (additional analysis)	see above (additional analysis)	Workplace-associated MSI:<br>Pre-post lost time injury count and rate (per hours worked), stratified by injury type (musculoskeletal versus non-musculoskeletal)<br>Work absence:<br>Pre-post working hours lost from injury - count and rate (per hours worked), stratified by injury type (musculoskeletal versus non-musculoskeletal)<br>Claims cost:<br>Pre-post total compensation costs from lost time injuries (count and rate), stratified by injury type (musculoskeletal versus non-musculoskeletal	see above (additional analysis)	see above (additional analysis)	see above (additional analysis)	**Univariate analysis (type: mean pre-intervention (SD) -> mean post-intervention (SD); (95% CIs of difference, p value of difference))**<br><br>Workplace-associated MSI:<br>Frequency rate (count per 10,000 hours worked):<br>Musculoskeletal injury rate 0.720(0.134) -> 0.299(0.116), (0.071 - 0.770) p < 0.05<br>Non-musculoskeletal injury frequency rate NS<br>Work absence:<br>Duration rate (logarithmic scale per 10,000 hours worked):<br>Musculoskeletal injury duration rate 2.200(0.177) -> 1.617(0.146); (0.172 - 0.995) p = 0.006<br>Non-musculoskeletal injury duration rate NS<br>Claims cost:<br>Claims cost rate (logarithmic scale per 10,000 hours worked)<br>Musculoskeletal injury claims cost rate 2.926(0.273) -> 1.816(0.198); (0.494 - 1.727) p > 0.01<br>Non-musculoskeletal injury claims cost rate NS

Carrivick 2005 [28]	see above (additional analysis): difference - cleaners group present in analysis, orderlies group (population control group) not present in analysis	see above (additional analysis): difference - cleaners group present in analysis, orderlies group not present in analysis	See above (additional analysis)	Workplace-associated MSI:<br>Pre-post lost time injury count and rate (per hours worked), stratified by injury mechanism (manual handling versus non-manual handling)<br>Work absence:<br>Pre-post working hours lost from injury - count and rate (per hours worked), stratified by injury mechanism (manual handling versus non-manual handling)<br>Claims cost:<br>Pre-post total compensation costs from lost time injuries (count and rate),	See above: difference - cleaners group present in analysis, orderlies group not present in analysis	See above (additional analysis)	See above (additional analysis)	**Univariate pre-post intervention analysis (mean rate before (SE) -> after (SE), 95% CI of difference, p value:**<br><br>Workplace-associated MSI:<br>Frequency rate per 10,000 hours worked (manual handling injuries): 0.619 (0.126) -> 0.185 (0.063), 0.164 - 0.704, $p < 0.01$<br>Non manual-handling injuries NS<br>Work absence:<br>Duration rate (logarithmic scale per 10,000 hours worked) (manual handling injuries): 2.090 (0.179) -> 1.584 (0.141), 0.128 - 0.886, $p < 0.01$<br>Non manual-handling injuries NS<br>Claims cost:<br>Claims cost rate (logarithmic scale per 10,000 hours worked) (manual handling injuries): 2.028 (0.194) -> 1.763 (0.189), 0.398 - 1.540, $p < 0.01$<br>Non manual-handling
Craib 2007 [30]	Quasi-experimental study (non-randomized experimental groups); 12 months follow-up	Community health workers from British Columbia, Canada: 6 organizations; nonrandomized; total n=648; organizations volunteered for inclusion	Checklist-based tool: summary sheet, hazard identification and assessment, biomechanics guide, handwashing technique, hazard prevention measures	Workplace-associated MSI:<br>Annual injury incidence proportion (all reported injuries; lost-time injuries) - operationalized as "time to first reported injury" and "time to first time-loss injury"	Intervention groups (5 organizations):<br>One or more of a combination of an education and training module, checklist-based risk assessment tool, lift equipment registry (total n=535)<br>Control group (1 organization):<br>No intervention provided. (n=171)	Resultant from Intervention group (checklist-based risk assessment tool): injury risk identification paired with control measure recommendations, suggestion of proper body mechanics	Workplace-associated MSI:<br>WorkSafeBC (provincial regulatory agency for injuries)	Workplace-associated MSI:<br><br>Univariate analysis:<br>Time to first reported injury: 13.9% annual injury (non-intervention program); 24.5% annual injury (intervention program); risk ratio 1.93 (95% CI 1.23 - 3.02; p-value < 0.05)<br>Time to first time-loss injury: 11.2% annual injury (non-intervention program); 6.9% annual injury (intervention program); risk ratio 0.61 (95% CI 0.35 - 1.07; NS)

Porru 2017 [36]	Quasi-experimental study (ITS); up to 7 years pre-intervention and 3 years post-intervention follow-up; yearly follow-up	Foundry workers from North/central Italy, categorized into 2 groups: ferrous foundries (n=22, 2750 individuals in 2007-08) and non-ferrous foundries (n=7, 710 individuals in 2007-08); foundries volunteered for inclusion	Ad-hoc risk assessment checklist: assessment of injury and near-miss data; provides foundry-specific recommendations for preventing similar accidents	Workplace-associated MSI:<br><br>Injury rate (incidence / population denominator)<br><br>Injury rate (injury density / hours-based rate)<br><br>Work absence:<br><br>Work absence rate (workdays lost per hours worked)	Intervention:<br>Use of risk assessment checklist and recommendations during telephone and on-site audits; improving worker access to occupational physician recommendations (including task adjustment); increased health surveillance; protective equipment prescriptioning (all foundries)<br>Control:<br>Pre-intervention period in each foundry (all [illegible]	Resultant from Intervention:<br>improvement of workplace maintenance, restriction of access of specific work areas, use of more suitable protective equipment, surveillance regarding use of protective equipment, increased access to workplace occupational physicians	Workplace-associated MSI:<br>Company-level databases of formally registered injuries of >3 days lost work<br><br>Work absence:<br>Company-level databases of formally registered injuries of >3 days lost work	Workplace-associated MSI:<br><br>Pre-post injury rate (incidence / population-based dedomination): 74% of initial injury rate following intervention (95% CI 57% - 95%)<br>NS for change in injury rate trend<br><br>NS for hours-based rate (rate or change in trend)<br><br>Work absence:<br><br>NS for work absence rate (rate or change in trend)
Rautianen 2004 [37]	Matched pair RCT; total of 3 years of follow-up	Farmers from a nine-county area in Iowa; n=125 for intervention and control cohorts for a total of 300 farms; participation based on respsnse from postage sent from US Postal mailing list	Certified Safe Farm (CSF)Checklist: identification and removal of farm hazards associated with illness and injury	Workplace-associated MSI:<br>Injury rate (individual count, quarterly injury rate (person-years)<br>Claims cost:<br>Mean injury cost (USD) and characteristics of insurance coverage (percent of injuries fully, partially, not covered by insurance)<br>Resource utilization:<br>Rate of injuries requiring hospitalization or professional care visit (person-year rate)<br>Work absence:<br>Rate of injuries requiring >1 day disability [illegible]	Intervention group:<br>CSF Checklist utilized during yearly on-farm safety reviews, health screening during clinic visits, informational and focus groups regarding intervention, $200 USD yearly payment (n=152)<br>Control group:<br>$75 USD yearly payment (n=164)	Resultant from Intervention group:<br>"Hazard identification and safe working methods" (not elucidated)	Workplace-associated MSI:<br>Annual occupational history forms; quarterly phone calls with standardized question formats; CSF-associated health calendars<br>Claims cost:<br>Quarterly phone calls with standardized question formats<br>Resource utilization:<br>Quarterly phone calls with standardized question formats<br>Work absence:<br>Unclear data collection mechanism (likely, a combination of resources used for collection of workplace-associated MSI [illegible]	Workplace-associated MSI:<br>Univariate analysis: Intervention effect: NS<br><br>Claims cost:<br>Mean injury cost of $163 USD<br>NS between intervention and control groups (p > 0.05)<br><br>Resource utilization:<br>Rate of injuries requiring hospitalization: NS between groups<br>Rate of injuries requiring professional care: NS between groups<br><br>Work absence:<br>Rate of injuries requiring >1 day disability (person-years): NS

Oude Hengel 2013 [35]	Cluster RCT; follow-up at 3, 6 and 12 months	Construction workers from 6 Dutch commercial construction companies (total n=293; comprised of 15 clusters)	"Quick scan questionnaire"	Musculoskeletal discomfort: Percent of individuals reporting symptoms for back, neck/shoulder, upper extremities, lower extremities<br>Sick leave: Dichotomous outcome: Count of individuals with 6+ days of sick leave in previous 6 months<br>Self-rated health status: Average score of SF-12 health survey	Intervention group: 1) Use of quick scan questionnaire by physiotherapist alongside 15-minute workplace inspection; 3 tailored recommendations made to reduce physical workload; discussion-based training session 4 months following initial intervention 2) Use of rest-break flow chart 3) 1 hour interactive training session for workplace empowerment (total n=171)<br>Control group: No intervention provided (total n=122)	Intervention group (quick scan questionnaire): three targeted recommendations by physiotherapist to reduce physical workload (work techniques, work methods, rest breaks)	Musculoskeletal discomfort: Self-report Dutch musculoskeletal questionnaire (DMQ) 4-point scale for pain and discomfort; operationalized as no symptoms / symptoms<br>Sick leave: Workplace databases from companies participating in the study<br>Self-rated health status: Self-report SF-12 health survey	Musculoskeletal discomfort: NS between intervention and control groups at 3, 6, or 12 months compared to baseline<br>Sick leave: NS between intervention and control groups at 3, 6, or 12 months compared to baseline<br>Self-rated health status: NS between intervention and control groups at 3, 6 or 12 months compared to baseline

Key: RCT=randomized controlled trial, HR=hazard ratio, CI=confidence interval, SD=standard deviation, NS=no statistical significance, OR=odds ratio, VDU=video display unit, USD=United States Dollars
